# TRAINING SENIOR VOLUNTEERS TO IMPLEMENT A GROUP MUSIC INTERVENTION FOR PERSONS WITH DEMENTIA

**DOI:** 10.1093/geroni/igz038.2452

**Published:** 2019-11-08

**Authors:** Silvia Orsulic-Jeras, Sarah Nicolay, Cristen Kane

**Affiliations:** 1 Benjamin Rose Institute on Aging, Cleveland, Ohio, United States; 2 RSVP of Lake County, Willoughby Hills, Ohio, United States

## Abstract

The Retired Senior Volunteer Program (RSVP) is a national program with many regional offices that offers a variety of volunteer opportunities for seniors. Research demonstrates the importance of volunteering and social participation on the well-being, health and longevity of older individuals. Further, studies of RSVP volunteers have demonstrated marked improvement in their perceived physical, mental health and quality of life (McDonald et al., 2013). Little evidence exists, however, supporting the effectiveness of using senior volunteers to assist with or even lead activities programming for persons with dementia (PWDs). Seniors Making Connections through Music, a group program designed to engage persons in the moderate to advance stages of dementia, included the training of 21 RSVP volunteers to lead or assist with the program. Volunteers were mostly female (95%) and educated (80% college/advanced degree), with more than half reporting professional or personal experiences with dementia (62%). Data were gathered through structured interviews and post-session debrief meetings. Findings suggest volunteers felt they were able to use existing knowledge and experience, received good and helpful supervision, and reported increased knowledge of dementia after volunteering for the group. Other results summarize: Volunteer satisfaction with program, role, tools and materials used, the program’s impact on the community, and willingness to volunteer for the program in the future. Programmatic features, training protocols, and the impact of using retired senior volunteers to strengthen program sustainability will be discussed. Volunteer testimonials and video demonstrations will also be shared.

